# Spectrum of *CFTR* mutations in Chechen cystic fibrosis patients: high frequency of c.1545_1546delTA (p.Tyr515X; 1677delTA) and c.274G>A (p.Glu92Lys, E92K) mutations in North Caucasus

**DOI:** 10.1186/s12881-019-0785-z

**Published:** 2019-03-21

**Authors:** N. V. Petrova, N. Y. Kashirskaya, D. K. Saydaeva, A. V. Polyakov, T.A. Adyan, O. I. Simonova, Y. V. Gorinova, E. I. Kondratyeva, V. D. Sherman, O. G. Novoselova, T. A. Vasilyeva, A. V. Marakhonov, M. Macek, E. K. Ginter, R. A. Zinchenko

**Affiliations:** 1 Federal State Scientific Budgetary Institution “Research Centre for Medical Genetics”, Moscow, Russia; 2 State Budgetary Institution “Maternity Hospital” of the Ministry of Healthcare of the Chechen Republic, Grozny, Chechen Republic Russia; 3 National Medical Research Center of Children’s Health, Federal State Autonomous Institution of the Russian Federation Ministry of Health, Moscow, Russia; 4 Department of Biology and Medical Genetics, 2nd Faculty of Medicine of Charles University Prague and Motol University Hospital, Prague, Czech Republic; 5 Federal State Budgetary Educational Institution of Higher Education “N.I. Pirogov Russian National Research Medical University” of the Ministry of Healthcare of the Russian Federation, Moscow, Russia

**Keywords:** Cystic fibrosis, *CFTR* mutations, Chechens, Russian Federation, Caucasus, c.1545_1546delTA (p.Tyr515X; 1677delTA)

## Abstract

**Background:**

Cystic fibrosis (CF; OMIM #219700) is a common autosomal recessive disease caused by pathogenic variants (henceforward mutations) in the cystic fibrosis transmembrane conductance regulator gene (*CFTR*). The spectrum and frequencies of *CFTR* mutations vary among different populations. Characterization of the specific distribution of *CFTR* mutations can be used to optimize genetic counseling, foster reproductive choices, and facilitate the introduction of mutation-specific therapies. Chechens are a distinct Caucasian ethnic group of the Nakh peoples that originated from the North Caucasus. Chechens are one of the oldest ethnic groups in the Caucasus, the sixth largest ethnic group in the Russian Federation (RF), and constitute the majority population of the Chechen Republic (Chechnya). The spectrum of *CFTR* mutations in a representative cohort of Chechen CF patients and healthy individuals was analyzed.

**Methods:**

Molecular genetic analysis of 34 *CFTR* mutations (representing approx. 80–85% of mutations in multiethnic CF populations of the RF) was performed in 32 CF patients from 31 unrelated Chechen families living in Chechnya. One hundred randomly chosen healthy Chechens were analyzed for the 15 most common “Russian” mutations. The clinical symptoms in Chechen CF patients with different *CFTR* genotypes were investigated.

**Results:**

High frequencies of c.1545_1546delTA (p.Tyr515X; 1677delTA) (52 out of 64 CFTR alleles tested; 81.3%) and c.274G > A (p.Glu92Lys, E92K) (8/64, 12.5%) mutations were found. Twenty patients were homozygous for the c.1545_1546delTA mutation, and eight were compound heterozygous for the c.1545_1546delTA and c.274G > A mutations. Three carriers of the c.1545_1546delTA mutation were also found in the cohort of 100 apparently healthy Chechens (frequency – 0.015). The c.1545_1546delTA and c.274G > A mutations are linked to the same haplotype (22–7–16–13) of intragenic Short Tandem Repeat markers, i.e., IVS1CA, IVS6aGATT, IVS8CA, and IVS17bCA.

**Conclusions:**

The distribution of *CFTR* mutations in the Chechen CF population is unique regarding the high frequency of mutations c.1545_1546delTA and c.274G > A (more than 90% of the mutant alleles). The c.274G > A mutation is associated with a less severe course of CF than that observed in c.1545_1546delTA homozygotes. Testing for these two variants can be proposed as the first step of CF DNA diagnosis in the Chechen population.

## Background

Cystic fibrosis (CF; OMIM # 219700) is a common autosomal recessive disease caused by pathogenic variants (henceforward mutations) in the *CFTR* gene (OMIM #602421). To date, over 2000 pathogenic, likely pathogenic, or benign *CFTR* variants have been identified [1]. The distribution of *CFTR* mutations varies widely in different populations [2]. Therefore, identification of the spectrum of the most common *CFTR* mutations in a given population can be used to optimize genetic counseling, foster reproductive choices, and facilitate implementation of mutation-specific therapies.

**Fig. 1:**
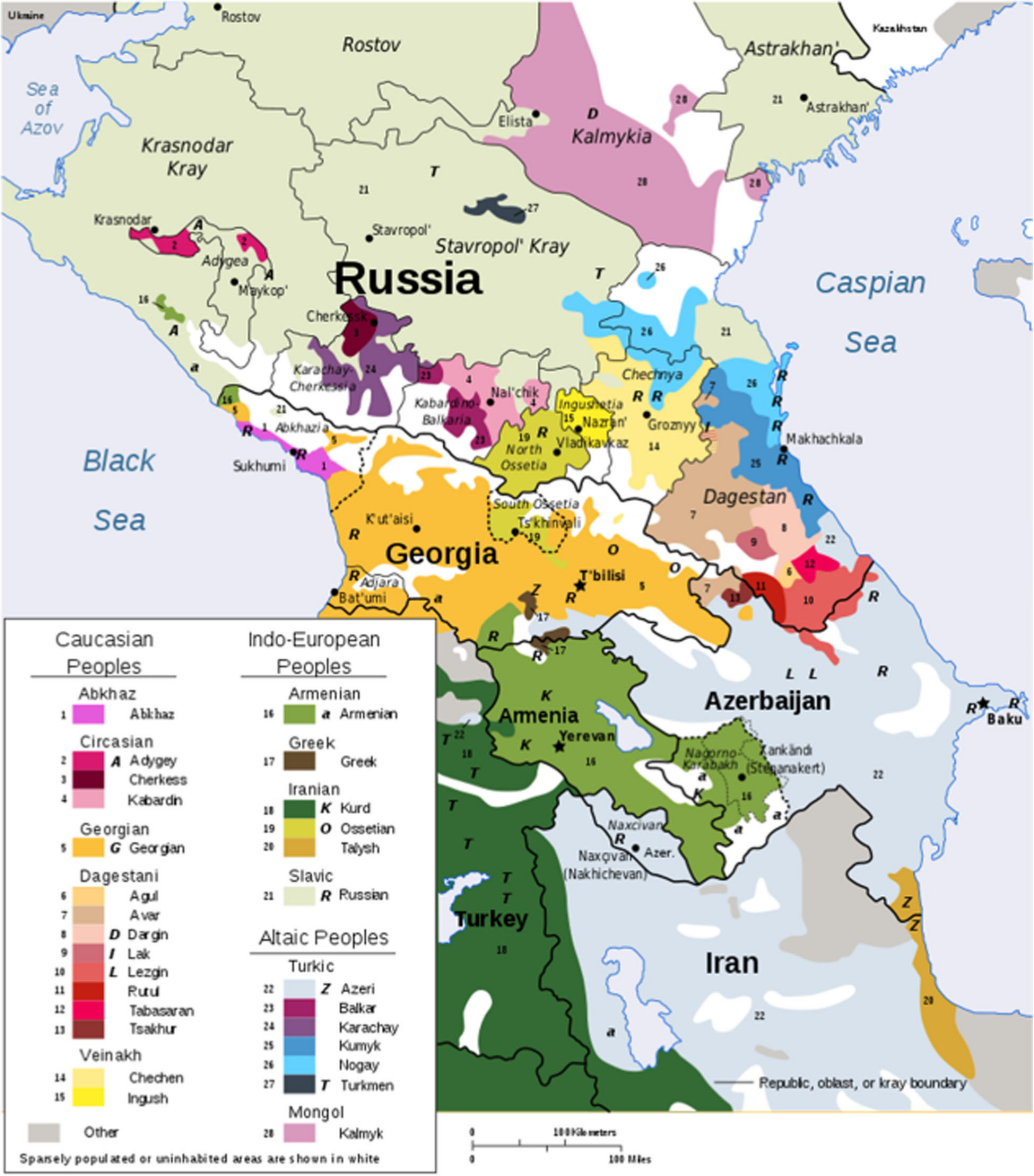
Ethnolinguistic groups in the Caucasus region. Wikimedia Commons is a collection of 49,714,663 freely usable media files to which anyone can contribute. https://commons.wikimedia.org/w/index.php?curid=2430263. Accessed 09 Jan 2019. This media licensed under the Creative Commons Attribution 2.5 License: https://creativecommons.org/licenses/by/2.5/

In this study, we have focused on the Chechen population that lives within the Chechen Republic of the Russian Federation (RF), located in the North Caucasus (see Fig. 1). Chechens are an ancient Nakh language speaking group that together with a related Ingush population are jointly termed “Vainakhs” [3, 4]. Both populations predominantly live in the Chechen and Ingush Republics of the RF (Fig. 1). Small portions of this distinct population also inhabit several neighboring districts of Dagestan and Georgia. Regarding their population size, the Chechens rank fourth in the Caucasus (after Azerbaijanis, Georgians, and Armenians) and sixth within the entire multiethnic RF (after Russians, Tatars, Ukrainians, Bashkirs, and Chuvash). The total number of Chechens around the world ranges between 1.5–2 million, with the majority of them (i.e. 1,344,122 according All-Russia Population Census) living in Chechnya proper [5].

Available historical and linguistic studies describe a single ancestral population that had been living on the northern slopes of the Great Caucasian Range (i.e., between the Caspian and Black sea regions) already several thousand years ago [6]. This presumed historic population had been associated with a Western Asian culture, distinct from the East Caucasus populations. Recent population genetic studies utilizing Y-chromosome haplotypes have demonstrated a robust genetic delineation of the Nakh language speaking people of Chechnya, Dagestan, and Ingushetia from other populations of the North Caucasus [7]. In this regard, Vainakhs are considered one of the oldest autochthonous Northern Caucasus ethnic groups [8]. The struggle for an independent Chechnya on the basis of the Nakh cultural-linguistic uniqueness ended in the 17^th^ and 18^th^ centuries CE (Common Era) when the Vainakh peoples became citizens of the former Russian Empire. In 1810, the Ingushs accepted Russian citizenship, followed by Chechens in 1859 [9].

In this study, we analyzed the spectrum of *CFTR* mutations in a representative cohort of Chechen CF patients and healthy individuals. According to the best of our knowledge, this is the first study of this population, which also has relevance for the strong Chechen diaspora in the RF and beyond.

## Methods

Our study included a representative cohort of 32 Chechen CF patients from 31 unrelated families. Except for a single case born in 1987, all patients were born between years 2004–2016. Patients and their families were self-reported ethnic Chechens living predominantly in the capital city Grozny. Cystic fibrosis was diagnosed based on consensus criteria for non-screened populations at the “Maternity Hospital” (Grozny, Chechnya) and in part at the Research and Clinical Department of Cystic Fibrosis of the Federal State Scientific Budgetary Institution “Research Center for Medical Genetics” or Federal State Autonomous Institution “National Medical Research Center of Children’s Health” of the Ministry of Health of the Russian Federation in Moscow.

We used a representative group of 100 unrelated apparently healthy Chechens as controls. The majority of them were also drawn from Grozny (67/100 of the entire control group), with the remaining volunteers originating from other regions of Chechnya. Ethnicity up to the third generation had been validated through a structured questionnaire filled under supervision. Healthy individuals, CF patients, or their legal representatives provided written informed consent for the study. This research project received approval from the Ethics Committee of the “Research Centre for Medical Genetics” (Moscow).

The “Wizard Genomic DNA Purification Kit” (Promega, USA) was used for DNA extraction from whole blood samples where EDTA was used as an anticoagulant. Initially, we examined the 34 most common *CFTR* mutations utilized for diagnosis of CF within the multiethnic RF that account for over 85% of all CF-causing mutations [10]. In-house molecular genetic methods previously described [11], including amplified fragment length (AFLP) and restriction fragment length (RFLP) polymorphism techniques were utilized to detect insertion/deletion variants and nucleotide substitutions, respectively. The panel of tested CF-causing mutations currently includes mutations: c.54-5940_273+10250del21kb (p.Ser18ArgfsX16; CFTRdele2,3), c.254G > A (p.Gly85Glu; G85E), c.262_263delTT (p.Leu88IlefsX22; 394delTT), c.274G > A (p.Glu92Lys; E92K), c.287C > A (p.Ala96Glu; A96E), c.350G > A (p.Arg117His; R117H), c.411_412insCTA (p.Leu138dup; L138ins), c.472dupA (p.Ser158LysfsX5; 604insA), c.489+1G > T (621+1G > T), c.1000C > T (p.Arg334Trp; R334W), c.1040G > C (p.Arg347Pro; R347P), c.1397C > G (p.Ser466X; S466X), c.1519_1521delATC (p.Ile507del; I507del), c.1521_1523delCTT (p.Phe508del; F508del), c.1545_1546delTA (p.Tyr515X; 1677delTA), c.1585-1G > A (1717-1G > A), c.1624G > T (p.Gly542X; G542X), c.1652G > A (p.Gly551Asp; G551D), c.1657C > T (p.Arg553X; R553X), c.2012delT (p.Leu671X; 2143delT), c.2051_2052delAAinsG (p.Lys684SerfsX38; 2183AA > G), c.2052_2053insA (p.Gln685ThrfsX4; 2184insA), c.2657+5G > A (2789+5A > G), c.3140-16T > A (3272-16T > A), c.3476C > T (p.Ser1159Phe; S1159F), c.3475 T > C (p.Ser1159Pro; S1159P), c.3535_3536insTCAA (p.Thr1179IlefsX17; 3667ins4), c.3587C > G (p.Ser1196X; S1196X), c.3691delT (p.Ser1231ProfsX4; 3821delT), c.3718-2477C > T (3849+10kbC-T), c.3816_3817delGT (p.Ser1273LeufsX28; 3944delTG), c.3844 T > C (p.Trp1282Arg; W1282R), c.3846G > A (p.Trp1282X; W1282X), and c.3909C > G (p.Asn1303Lys; N1303K)) [10].

Subsequently, for a case in which one *CFTR* mutation remained unidentified we carried out direct Sanger DNA sequencing of the entire *CFTR* coding region, including adjacent splice sites and the 3′-untranslated *CFTR* region [10]. Positive cases were confirmed in parents to establish their linkage phase. Random controls were analyzed for the 15 most common “Russian” mutations: c.54-5940_273+10250del21kb (p.Ser18ArgfsX16; CFTRdele2,3), c.262_263delTT (p.Leu88IlefsX22, 394delTT), c.274G > A (p.Glu92Lys, E92K), c.287C > A (p.Ala96Glu, A96E), c.411_412insCTA (p.Leu138dup; L138ins), c.1000C > T (p.Arg334Trp; R334W), c.1521_1523delCTT (p.Phe508del, F508del), c.1519_1521delATC (p.Ile507del, I507del), c.1545_1546delTA (p.Tyr515X; 1677delTA), c.2012delT (p.Leu671X, 2143delT), c.2051_2052delAAinsG (p.Lys684SerfsX38, 2183AA > G), c.2052_2053insA (p.Gln685ThrfsX4; 2184insA), c.3691delT (p.Ser1231ProfsX4; 3821delT), and c.3846G > A (p.Trp1282X; W1282X).

Four intragenic short tandem repeats (STR) (IVS1CA, IVS6aGATT, IVS8CA, and IVS17bCA) were examined as previously described [12]. STR haplotypes were established by segregation analysis of given *CFTR* alleles within CF families. STR haplotype frequencies in healthy samples were calculated using ARLEQUIN version 3.5 software [13].

To assess the course of the disease in Chechen CF patients with different *CFTR* genotypes, the following key parameters were taken into account: the patient’s age at the last examination, the age at diagnosis, sweat test (chlorides, mM/L), body mass index (BMI) (kg/m^2^), spirometry parameters FEV_1_ (% predicted) and FVC (% predicted), pancreatic insufficiency (fecal elastase 1 (< 200 μg/g)), complications (meconium ileus, liver cirrhosis (with/without portal hypertension), CF-related diabetes mellitus, allergic bronchopulmonary aspergillosis (ABPA), and chronic sino-bronchial colonization by *S. aureus, P. aeruginosa, B. cepacia complex, Achromobacter spp, S. maltophilia,* and nontuberculous mycobacteria (NTM) including Gram-negative microflora.

Statistical analysis was performed using the STATISTICA 8.0 program. To compare observed categorical variables, the Fisher test was used, while for quantitative tests the Mann-Whitney test was utilized. Results were considered as significant when *p* ≤ 0.05.

## Results

In the Chechen Republic, 33 Chechen CF patients are officially registered. The prevalence of CF was 2.455 per 100,000 Chechens in 2017. Nearly all of the known CF patients residing in Chechnya (32/33) were analyzed.

Analysis of the 34 common *CFTR* mutations in the Russian multiethnic CF populations revealed a particularly high frequency of two mutations in the Chechen population: c.1545_1546delTA (p.Tyr515X; 1677delTA) – 52/64 *CFTR* alleles (81.3%), and c.274G > A (p.Glu92Lys, E92K) – 8/64 alleles (12.5%) (Table 1). Twenty patients were homozygous for c.1545_1546delTA (p.Tyr515X; 1677delTA), while 8 were compound heterozygous for the c.1545_1546delTA (p.Tyr515X; 1677delTA) and c.274G > A (p.Glu92Lys, E92K) mutations. In addition, c.287C > A (p.Ala96Glu, A96E), c.1000C > T (p.Arg334Trp; R334W), c.3846G > A (p.Trp1282X; W1282X), and a novel variant c.3925_3936delCAGTGGAGTGAT (p.Trp1310_Gln1313del) were identified once in each case (Table 1).

**Table 1:** Distribution of *CFTR* gene mutations in Chechen CF patients

*CFTR* genotypes	Number of patients (*n* = 32)	Frequency
c.[1545_1546delTA];[1545_1546delTA] (p.[Tyr515X];[Tyr515X]; 1677delTA/1677delTA)	20	0.625
c.[1545_1546delTA];[274G > A] (p.[Tyr515X];[Glu92Lys]; (1677delTA/ E92K)	8	0.251
c.[1545_1546delTA];[287C > A] p.[Tyr515X];[Ala96Glu]; 1677delTA/A96E)	1	0.031
c.[1545_1546delTA];[1000C > T] (p.[Tyr515X];[Arg334Trp]; 1677delTA/R334W)	1	0.031
c.[1545_1546delTA];[3846G > A] (p.[Tyr515X];[Trp1282X]; 1677delTA/W1282X)	1	0.031
c.[1545_1546delTA];[3925_3936delCAGTGGAGTGAT] (p.[Tyr515X];[Trp1310_Gln1313del])	1	0.031
*CFTR* alleles	Number (*n* = 64)	Frequency
c.1545_1546delTA (p.Tyr515X; 1677delTA)	52	0.8130
c.274G > A (p.Glu92Lys, E92K)	8	0.1250
c.287C > A (p.Ala96Glu, A96E)	1	0.0155
c.1000C > T (p.Arg334Trp; R334W)	1	0.0155
c.3846G > A (p.Trp1282X; W1282X)	1	0.0155
c.3925_3936delCAGTGGAGTGAT (p.Trp1310_Gln1313del)	1	0.0155

In the control group of 100 randomly chosen Chechen individuals, 3 carriers of the c.1545_1546delTA mutation were detected (1.5%), while none of the 14 remaining common “Russian” *CFTR* mutations were detected.

The c.1545_1546delTA and c.274G > A mutations were present on a single intra-*CFTR* short tandem repeat (STR) haplotype “22–7–16–13” (as a sequence of IVS1CA, IVS6aGATT, IVS8CA, and IVS17BCA STR markers), this suggesting their common ancestral origin. Interestingly, this haplotype was also the most common in Chechen controls (47.5%).

To compare the clinical course of CF in the studied cohort, the patients were divided into the two most prevalent groups: 17 homozygous for c.1545_1546delTA (Group 1), and 8 compound heterozygotes for c.1545_1546delTA and c.274G > A variants (Group 2). We did not find significant differences in the patient’s age at last clinical examination, age at diagnosis, sweat Cl^−^ concentrations, or BMI values. None of the most common before-mentioned complications (meconium ileus, liver cirrhosis, diabetes, polyposis) were revealed in either group. Significant differences were observed only in terms of pancreatic insufficiency in that all patients from Group 1 had fecal elastase 1 concentrations below 50 μg/g, while all patients from Group 2 had concentrations over 200 μg/g (*p* < 0.0001), this indicating lower degree of pancreatic exocrine dysfunction associated with the presence of c.274G > A. Similarly, the proportion of patients with chronic *P. aeruginosa* lung colonization was significantly higher in Group 1 compared to Group 2 (69.0% vs. 14.0%, respectively; *p* = 0.024). The differences in the other studied microorganisms were not significant. Overall, the presence of the c.274G > A mutation is associated with less severe course of the disease than in the c.1545_1546delTA homozygotes (Table 2).

**Table 2:** Comparison of two groups of Chechen CF patients

		Group 1 (*n* = 17)	Group 2 (*n* = 8)	
*CFTR* genotype		c.[1545_1546delTA];[1545_1546delTA] (p.[Tyr515X];[Tyr515X]); (1677delTA/1677delTA)	c.[1545_1546delTA];[274G > A] (p.[Tyr515X];[Glu92Lys]); (1677delTA/E92K)	*p*-value
Age at last clinical examination (yrs)		5.66 ± 8.28 (0.29÷31.46)	4.53 ± 4.13 (0.92÷11.92)	> 0.05
Age at diagnosis (yrs)		1.66 ± 0.91 (0.00÷20.18)	1.07 ± 0.91 (0.16÷3.00)	> 0.05
BMI (kg/m^2^)		14.93 ± 3.12 (12.30÷24.88)	15.92 ± 2.55 (13.00÷21.00)	> 0.05
Sweat chloride (mM/L)		120.25 ± 36.27 (100.00÷134.00)	120.25 ± 15.62 (100.00÷134.00)	> 0.05
FEV_1_ (% predicted)		82.66 ± 26.85 (52.00÷102.00)	91.50 ± 2.12 (90.00÷93.00)	> 0.05
FVC (% predicted)		93.66 ± 27.09 (68.00÷122.00)	91.50 ± 2.12 ((90.00÷93.00)	> 0.05
Meconium ileus		0	0	
Liver cirrhosis		0	0	
CF-related diabetes mellitus		0	0	
Fecal elastase 1 concentration	≥200 μg/g	0	8	< 0.0001
	*<* 200 μg/g	17	0	
*S. aureus* lung colonization		44%	14%	> 0.05
*P. aeruginosa* lung colonization		69%	14%	0.024

## Discussion

To the best of our knowledge, this is the first study on the distribution of *CFTR* mutations in the Chechen population (Fig.1). We have provided evidence that the c.1545_1546delTA and c.274G > A mutations (as validated by the www.cftr2.org database) account for the majority of *CFTR* mutations in this population. These CF alleles very likely have a common origin, since they are residing on a common population-specific intra-*CFTR* STR haplotype that probably increased in frequency due to genetic drift.

The c.1545_1546delTA mutation was previously found to be common in populations neighboring or with historic links to the greater Black Sea region [2] (e.g., Bulgaria, Romania, Greece, Cyprus, and Turkey, including Northern Iran and Georgia [14]). It was also identified in other ethnic groups from the Northern Caucasus region (i.e., Ingush, Armenians, Ossetians, Dagestanis, etc.), albeit only in a small number of CF patients examined [10]. The fact that the c.1545_1546delTA mutation is found in autochthonous ethnic groups of the Caucasus region (Chechens, Ingush, Georgians, Armenians) and is linked to a single haplotype may point to a single source of origin (or penetration) of this mutation into the Caucasus region, and differences in frequencies seen in various populations may be due to gene drift.

Interestingly, the c.274G > A mutation was found at the highest frequency in Chechen CF patients (12.5%). This CF allele was previously found in Turkey [2], but also in Chuvash CF patients living in central parts of the RF (55.6%) [15]. According to the Russian Cystic Fibrosis Patient Registry (RCFPR), the prevalence of this allele in patients in the Volga-Ural region is 6.88% (Chuvash Republic – 53.19%, Udmurt Republic – 6.76%, Tatarstan – 2.38%, Bashkortostan – 1.37%, Samara region – 3.06%, Perm – 0.75%, and Orenburg regions – 1.96%), including the Khanty-Mansi autonomous region (Yugra) – 3.85%, as well as sporadically in many other RF regions [16]. Given its population distribution, the c.274G > A mutation could be associated with the historical resettlements of Turkic-speaking peoples in regions of RF metioned above, including their migrations to the Caucasus and greater Black Sea geographical area.

Although the c.3846G > A mutation was found at a very high frequency in the neighboring Karachay-Cherkessia (Fig.1) [11], it was only sporadically observed in Chechens, thereby substantiating the strong genetic delineation of Vainakh populations from other ethnic groups residing in the Northern Caucasus. The penetration of the c.3846G > A mutation into the territory of the Northeast Caucasian region could be related to the migration of Jews from Byzantium through the northern Black Sea region or Georgia in the early Middle Ages or from Persia (Iran) in the late Middle Ages [11].

The c.287C > A mutation was previously described in Turkish CF patients, where its frequency is at 2.6% [2]. In the RF this mutation was also found in 2 patients from Dagestan, which is bordering Chechnya (Fig.1).

Finally, the c.1000C > T mutation is in CF populations from the greater Mediterranean region, such as those from southern France (1.2%), Greece (1.1%), Portugal (0.7%), and Spain (1.2%) [2], while in the RF it was sporadically observed in various ethnic groups at the frequency of 0.8% [16].

The comparison of key clinical parameters in the two groups of Chechen patients with different genotypes demonstrated that the allele c.274G > A is associated with higher residual pancreatic function and lower chronic lung colonization with pathognomonic microorganisms, in accordance with the data of the CFTR2 database [1].

## Conclusions

The distribution of *CFTR* mutations in the Chechen CF population is unique in terms of the high frequency of mutations c.1545_1546delTA (p.Tyr515X; 1677delTA) and c.274G > A (p.Glu92Lys, E92K), which account for more than 90% of the mutant alleles in the studied ethnic group. Testing of the two CF-causing mutations is thus recommended for Chechen CF patients, since it allows identification of one or both mutant *CFTR* alleles in more than 99% of patients suspected of being affected by CF. Furthermore, we have confirmed the genetic delineation of the Chechen population from other ethnic groups of the Northern Caucasus (e.g. by low prevalence of the c.3846G > A mutation, which is dominant in adjacent Karachay-Cherkessia; Fig.1), as well as the role of historic migrations of Turkic-speaking peoples from Central Asia to the Northern Caucasus with the c.274G > A mutation very likely being their “marker” [17]. Analysis of genotype–phenotype correlations in two groups of Chechen CF patients (i.e. c.1545_1546delTA homozygotes versus c.1545_1546delTA/c.274G > A compound heterozygotes) demonstrated that the presence of the c.274G > A mutation is associated with generally less severe course of the disease. Our data will improve genetic counselling and provide a basis for the introduction of mutation-specific therapies in the future.

## Abbreviations

BMI: Body mass index
CF: Cystic Fibrosis
*CFTR*: Cystic Fibrosis Transmembrane Regulator
CFTR2: The Clinical and Functional TRanslation of CFTR (CFTR2) (http://cftr2.org)
DNA: Deoxyribonucleic acid
FEV_1_: Forced expiratory volume at 1 s. (spirometric parameter)
FVC: Forced vital capacity (spirometric parameter)
RF: Russian Federation
STR: Short Tandem Repeats
